# Communication training for centre-based carers of children with severe or profound disabilities in the Western Cape, South Africa

**DOI:** 10.4102/ajod.v1i1.10

**Published:** 2012-09-21

**Authors:** Martha Geiger

**Affiliations:** 1 Centre for Rehabilitation Studies, University of Stellenbosch, South Africa

## Abstract

The purpose of this paper is to provide a preliminary, qualitative review of an approach to training centre-based carers in supporting basic communication development and providing communication opportunities for the children with severe and profound disabilities in their care. In South Africa, these children are often the most neglected in terms of planning and providing appropriate interventions. For those with severe *communication* disabilities, an additional lack is in the area of the basic human right to meaningful interactions and communication. Sustainable strategies to provide opportunities for basic communication development of these children are urgently sought. Several effective international and local *parent* training programmes have been developed, but the urgent need remains to train *centre-based carers who are taking care of groups of diversely disabled children in severely under-resourced settings*. Non-profit organisations (NPOs) have been exploring practical centre-based approaches to skills sharing in physical rehabilitation, activities for daily living, feeding and support for basic communication development. As a freelance speech therapist contracted by four NPOs to implement hands-on training in basic communication for centre-based carers of non-verbal children, the author describes a training approach that evolved over three years, in collaboration with the carers and centre managements. Implications for training (for speech therapists and for community-based rehabilitation workers) and for further research are identified.

## Introduction

Communication with others is an activity that defines our humanity. Yet, in spite of policy and legislative frameworks emphasising communication as a basic human right (Department of Health 2000; United Nations 2008), it is often overlooked and underestimated as a basic human need. The motivation for writing this paper comes from the author’s observations of how children with severe or profound physical, intellectual and/or communication disabilities, attending special care centres in under-resourced settings, are often deprived of this basic right due to multiple factors, and yet, how do-able steps can facilitate meaningful interaction and functional communication opportunities.

This review is a reflection on an evolving and dynamic skills-sharing approach and a preliminary step in the quest for a model of best practice for training and empowering carers of groups of diversely disabled children in formal and informal special care centres in the Western Cape, South Africa.

### Problem statement

Prevalence data on children with severe disabilities in South Africa are sketchy (Schneider and Saloojee 2007). However, reports confirm increases in severe disabilities in children, secondary to pre-, peri- and postnatal birth complications (ACPF 2011; WHO 2011). These are further compounded by the prevailing social and environmental conditions, including poverty, limited access to health and rehabilitation services, as well as the effects of HIV and AIDS (ACPF 2011; WHO 2011). Furthermore, these children, and especially those with severe and profound intellectual disabilities, are often the most neglected in terms of planning and providing appropriate interventions and services (ACPF 2011; Ransom 2009; Saloojee *et al*. 2006; WHO 2011). In South Africa, children with severe or profound disabilities have been excluded from educational inputs for many years (Western Cape High Court 2011) and regular, formal speech therapy services (limited to the tertiary hospitals in the area) are often not accessible to these children. Non-profit organisations (NPOs) have attempted to alleviate this situation by providing much needed but often fragmented ad hoc speech (and other) therapy services, and training and support of carers in the special care centres in and around Cape Town.

**The policy environment – in brief:** International and local policies provide a strong directive for adopting rights-based approaches to service delivery for people (including children!) with disabilities. At the global level, the United Nations Convention on the Rights of Children emphasises the rights of all children with disabilities to access all the help they need (United Nations 1989; Article 23). Furthermore, the United Nations Convention on the Rights of Persons with Disabilities (UNCRPD) elaborates on the rights of persons with disabilities in detail, including the right for persons with disabilities to have access to communication and to participate in their communities (United Nations 2008). South Africa has formally ratified the UNCRPD and therefore accepted it as legally binding. In terms of international education policies, South Africa had also signed the earlier ‘Salamanca Statement and Framework for Action on Special Needs Education’ (UNESCO 1994) – committing to the principle of education *for all*.

At a national level, South Africa has some of the most progressive human rights-based legislation in the world, yet its implementation is often lacking (ACPF 2011; Dube 2006; Ogot, McKenzie & Dube, 2008; Schneider & Saloojee 2007). National policies detail the vision of a ‘society for all’ and the Integrated National Disability Strategy (INDS), serves as a framework for the integration of disability issues in *all* governmental development strategies (Office of the Deputy President of South Africa 1997).

However, children with severe disabilities, including those with a severe or profound intellectual disability and an IQ of less than 35, have been formally excluded from fundamental educational and training inputs for many years (WCFID 2011; Western Cape High Court 2011; Wood *et al*. 2009). Special care centres for these children have not been included in any form of strategic planning or budgetary provision for appropriate educational services or even human resources for basic stimulation and training provision (Wood *et al*. 2009). Spearheaded by the Western Cape Forum for Intellectual Disability (WCFID), efforts to challenge and change this legislation have finally reached a victory in the High Court of South Africa in November 2010 (Western Cape High Court 2011). While audits are currently underway to assess the exact needs and possibilities, the implementation of this ruling will take time.

On another front, the National Rehabilitation Policy (Department of Health 2000) addresses the provision of assistive devices in South Africa, including augmentative and alternative communication (AAC) devices. Globally, technological and other developments in the specialised field of AAC have delivered revolutionary possibilities for people with severe communication disabilities (Alant 2005; Beukelman and Mirenda 1998; Schlosser 2003). However, the translation of these technological developments and other evidence-based AAC strategies into functional communication is still decades away in many under-served and low income communities, where policy rollouts, financial and human resources in terms of skills and numbers are lacking (McConkey 2005). This is clearly evident in the population of children with severe or profound disabilities in the extensively disadvantaged contexts of the Western Cape. In the meantime, at grassroots level, there is an urgent need for awareness-raising and enskilling of centre-based carers, to provide the means and moreover the opportunities for the most basic, pre-verbal interactions and non-verbal communications for children with severe and profound disabilities.

**The situation at grassroots level:** A repeatedly observed situation in disadvantaged areas of the Western Cape is that mothers of children with severe disabilities cannot seek employment as they cannot find day care facilities for their children. Very few mainstream crèches and day care centres accept children with disabilities, especially severe disabilities. This is sometimes due to negative, albeit unknowing, attitudes and pressures of other parents, not to accept children with disabilities due to the additional care they require and/or persistent beliefs that disabilities (or at least their causes) are contagious (Chataika 2011; Duncan *et al*. 2009; Ingstad & Reynolds-Whyte 1995). Whilst there are some encouraging examples of mainstream crèches accommodating children with disabilities and of other parents embracing and assisting such children and their families, this is still the exception rather than the norm. Several *special care centres* have thus been started by mothers of disabled children themselves who have extended their care to other disabled children. Some of these community-based initiatives, which began as small informal centres, have developed into larger, more formal special care centres. As the centres grow and more help is needed, other unemployed mothers who seek care for *their* children with disabilities sometimes begin working as volunteers or become carers at such centres.

These community-based initiatives usually struggle through years of self-funding or are run on a portion of the Care Dependency Grants^1^ of those attending children who receive them. Even after formalisation and qualification for formal subsidies, the available funding in the special care centres only covers minimal human resources. The observed norm is disproportionately high child-to-staff ratios and poorly paid staff or volunteers, who are mostly untrained, but include enormously motivated and capable mothers of children with disabilities themselves. These human resources are insufficient to implement even the most basic individual intervention plans, as carers are in ‘survival mode’ and barely manage with the basic care needed. In spite of extremely difficult conditions such as large, mixed-impairment groups and cramped and under-resourced facilities, carers continue to provide excellent and loving basic care to the children – often for no or below-minimum wages.

It is not uncommon to find informal centres where children with severe disabilities are treated as *sick patients*, confined to high-sided cots, fed, cleaned and medicated – albeit with the utmost care – very much in line with ‘medical model’ thinking (Duncan *et al*. 2009; Ingstad & Reynolds-Whyte, 1995; Ross & Deverell 2004). There are no therapeutic inputs or stimulation, nor opportunities for the children to explore, play or interact with others at their respective levels of ability. However, they need opportunities and support to enable them to develop to their utmost potential and to achieve the best quality of life possible.

Information from *parent* training programmes such as the internationally renowned Hanen Programme (Pepper & Weitzman 2004) and the Portage Project (Sampon & Wollenburg 1998), as well as local research with primary caregivers in nuclear family situations (Popich, Louw & Eloff 2007) provided helpful content but limited application for caregiver training in these centre-based group contexts. Likewise, research about centre-based caregiver training in well-resourced, developed country contexts with low child to carer ratios (Girolametto, Weitzman & Greenberg 2003), or even more local programmes for specific disabilities (Hambisela, n.d.) were found to have limited application in the specific local contexts of under-resourced and understaffed centres, with previously untrained carers caring for large groups of children with diverse disabilities, ages and levels of functioning. However, provisional local research results on addressing the *social context*, or human interactions within the environment of residential settings for orphaned and vulnerable (not necessarily disabled) children, through caregiver training, are of great interest and hold much promise (Koch & Franzsen 2012; Koch & Kok 2012).

In the area of basic communication, the need is for carers to be empowered to facilitate and support meaningful interactions and optimal communication development – albeit mostly non-verbal – for the children in their care. Several of the NPOs who have been backing grassroots special care centres for children with severe disabilities, are supporting the implementation and exploration of training approaches for such basic communication.

This preliminary, descriptive review of what has been helpful and what has not, is a small step in the much needed, more formal enquiries into questions of effective, relevant and sustainable centre-based communication training interventions.

## Methods

Reflective practice (Schön 1995) and iterative or self-generating cycles of action (Chambers 2010; Denzin & Lincoln 2000), comprising reflection and adjustment to planning and implementation of communication training sessions across three years, have facilitated some preliminary conclusions and tentative recommendations.

Funding restraints and the number of centres that requested training in basic communication development, meant that the training was offered at a total of sixteen special care centres, in cycles of three to six three-hour sessions. Where possible all the carers in a centre were included in the training, but emergency needs including washing children, cooking and cleaning, etc. meant that carers often came and went during the course of a session. All the carers were female, with ages ranging from 18 to beyond retirement age (65+).

The challenge for this therapist offering the training in basic communication was to utilise the limited time in the most effective and efficient way. Crucial considerations included the lack of physical resources (e.g. space and equipment) in these centres; the diverse mix of impairments represented in each centre; the wide age range of children often in a single group (three to 18 or even 21); the high child-to-carer ratios (often up to 10:1, and 18:1 in one situation); the labour-intensive basic, physical care needs (feeding, cleaning, frequent medication etc.); the challenges facing the biological mothers in carrying out individual stimulation programmes at home; and the fact that the majority of the centre-based carers have no formal, basic training.

The emphasis and priorities of this training therefore focused upon what would be most relevant to as many of the children’s needs as possible, what could be implemented by the already overstretched carers in a sustainable manner, and what would enhance the children’s functional interactions, basic communication and quality of life.

### Structure and content of the on-site communication training

The previously implemented individual therapy model, that is, therapists going into the centres and doing *ad hoc* hands-on work with carers and individual children, was clearly not effective or sustainable: records indicated a trend of more assessments than actual therapy. As a result, a participatory learning and action (PLA) approach was followed (Chambers 2007; Hope & Timmel 1995, 1999; Hartley *et al*. 2005). The structure of the on-site communication training sessions with carers evolved by using the cyclical process of implementation, reflection and/or evaluation, and adjusted implementation (Chambers 2007; Hope & Timmel 1995, 1999; Hartley *et al*. 2005). A combination of basic communication theory and hands-on skills transfer was found to be most effective, with the following sections and approximate time allocations within each session:

- Opening challenge
- Tutorial (opening challenge plus tutorial made up about 20% of session time)
- Hands-on skills transfer with carers and children (about 70% of session time)
- Wrap-up with questions, self-evaluation and next steps (about 10% of session time).

**Opening Challenge:** The purpose of the opening challenge was to focus the group and to establish the training needs and priorities of the carers. This time was guided by the three Freirian questions (Freire 1970; Hope & Timmel 1995, 1999; Hartley *et al*. 2005): *‘What is the problem?’ ‘Why is it a problem?’* and *‘What can we do about the problem?’* The focus was thus upon the carers’ perceived priority problems, challenges and possible solutions.

**Tutorial:** The tutorial section included teaching topics such as ‘*Basics of communication development’, ‘Diverse communication disabilities’* and ‘*Participatory communication strategies’*. There were slight variations according to the self-identified needs of each centre and different groups of carers. The format was an interactive teaching approach focussing upon examples contributed by the carers present.

#### Tutorial topic 1: Basics of communication development

**FIGURE 1:**
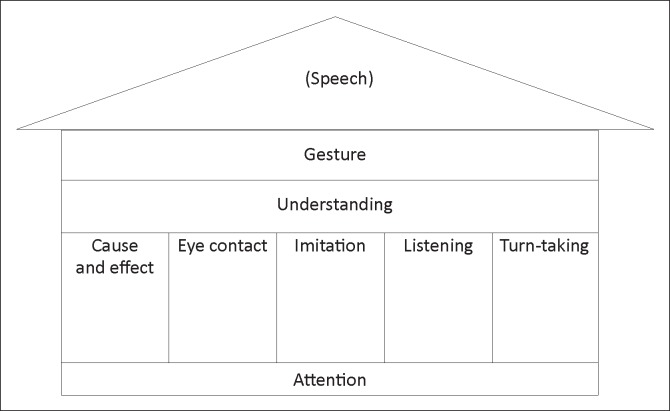
‘The Communication House’ adapted from *Let’s communicate* (WHO 1997).

This introduction followed the principles as presented in the handbook, *Let’s communicate* (WHO 1997). It emphasised the importance of developing the prerequisite pre-verbal skills of attention, cause and effect, eye contact, imitation, listening, turn-taking, understanding and all forms of non-verbal communication, including the use of the voice in the absence of speech, body language, hand or eye-pointing and gestures. The image of the communication house (Figure 1) needing a strong foundation (pre-requisite attention for communication) struck a chord with most carers. The support and development of these foundational skills was also emphasised in the hands-on skills sharing sessions (described further on).

Furthermore, an awareness of the frequent and close causal relationship between problems related to these pre-verbal communication skills (e.g. ‘attention’) and challenging behaviours was variably explored, depending on carer needs and readiness in different centres. One success story involved a carer in a particularly noisy centre, who implemented a simple strategy with a particularly disruptive child: she would speak the child’s name, pause to wait for eye contact, – smile and then give the child a simple instruction and verbal praise upon completion: all in a soft voice. This encouraged the child to attend to the carer much more than before because he enjoyed the interaction and the verbal reinforcements so much.

#### Tutorial Topic 2: Diverse communication disabilities

Children with severe or profound disabilities make up a very heterogeneous population in terms of communication strengths and weaknesses. Some key differences in *functional* communication components (WHO 2001) were explored with the carers. This was crucial in identifying strategies for managing their groups of very diversely disabled children. For example, different communicative support and opportunities were needed for:

- children with severe cerebral palsy, who could not speak or use gestures but could understand almost everything and were extremely motivated to communicate
- children with autistic behaviours whose ability to connect with others was the most affected area and often resulted in challenging behaviours
- children with Down syndrome whose strengths included their desire to connect with (and please) others and their effective use of a limited repertoire of a few clear utterances for many meaningful, purposeful interactions.

While the above is an oversimplification, it was useful in helping carers to understand why children differed so much in their needs of stimulation and how they could be grouped for optimal stimulation and communication opportunities. For example, one carer had a group of eight children, six with severe intellectual disabilities, relatively strong physical abilities and very low abilities to connect with others (i.e. with autistic tendencies); and two girls with much higher levels of understanding and a great desire to communicate, but limited means of expression due to severe cerebral palsy and no speech nor sufficient motor abilities to gesture or sign. Grasping these differences, the carer proceeded to position the two girls next to each other, where she could direct higher level visual stimulation and expectations to them together and watch for eye blink and other body language responses from them more easily than when they were placed among the less communicative, more physically active and distracting peers, where she often missed the two girls’ communication attempts. Moreover, the two girls could develop a real friendship through their very astute perception of one another’s subtle non-verbal communication.

#### Tutorial Topic 3 – Participatory communication strategies

The importance of inclusion and participation as applied to their children was discussed with the carers. Two issues regularly became clear:

- Challenging behaviour, secondary to intellectual disability, and often the child’s only means of communication (Beukelman & Mirenda, 1998), was the most frequently given reason why parents and carers could or would not include children in family or social activities. The links between developmental delays in preverbal skills (such as attention, listening skills and most especially, turn-taking) and challenging behaviours were explored with carers and the children in their care. Practical strategies such as reducing background noise levels, and consistent cause and effect systems like ‘time out’ were practiced to support these preverbal skills, the lack of which often caused frustration and challenging behaviours – which contributed to the vicious cycle of exclusion and more asocial behaviours.
- Even within the centres, some children were previously regularly excluded from group games or activities, and their inclusion and participation was addressed. Examples included those with the most severe physical impairments, who were wheelchair or buggy users or positioned in standing frames or even side-lyers, and were previously thought to be incapable of participating in ball and other group games. The need for these children to have opportunities to play (e.g. the excitement of having a turn to receive and pass on the ball like everyone else) was emphasised. Likewise, children with severe intellectual disability and related challenging behaviours (such as ‘autistic’ behaviours and/or attention deficits and hyperactivity) were previously excluded on the grounds that it was too difficult to include them or that they were thought to ‘prefer to be on their own’. Their need to learn to focus, attend, listen, wait for their turn etc. was elaborated by referring back to the ‘Communication House’ presented earlier (WHO 1997). Specific strategies to include all these children in the circle activities, and to facilitate the participation of all, were problem-solved collaboratively and then practiced in the hands-on session, outlined below.

**Hands-on skills transfer with carers plus children:** Time allocated to hands-on skills sharing was aimed at practicing skills that had first been discussed during the tutorials, and included:

- Morning rings, greeting and naming rituals emphasising individual children’s names and getting their attention and encouraging them to participate vocally or with basic gestures. These were already widely, although in some centres irregularly, practiced. Therefore, value-added aims such as enhancing active participation, developing attention, listening skills and turn-taking abilities during these activities were applied and practiced.
- Song circles: These had previously also been implemented inconsistently. Building upon these, the repetition of a small number of known and loved songs (preferably with some actions and/or a repetitive chorus and clapping) was found to encourage participation more than a wider ever-increasing repertoire of songs. Carers were made aware of the value and enjoyment of repetition for the children in their care. The common tendency to reinforce only responsive children was counteracted by strategies that supported the active participation and enjoyment of each and every child according to the child’s ability. It was repeatedly observed that carers were surprised by ‘unresponsive’ children’s capacity for enjoyment during these activities. In line with Sameroff and Fiese’s well-known research on transactional regulation (2000), it was also observed that the reciprocity between child enjoyment and carer enjoyment contributed to the sustainability of these inclusive, multi-goal activities.
- Inclusive ball games: These were practiced as a strategy to help carers to meet different goals (such as attention, turn taking etc.), for individual children while engaging a whole group of diversely disabled children in a single activity. Other, similar group games were also encouraged as an empowering strategy for carers who, due to the staffing shortages, often had to cope alone with a group of up to ten (or even 18!) diversely disabled children. Those who were physically and intellectually stronger could be interspersed between those in buggies and/or those temporarily restrained on children’s chairs to participate better. The task of waiting for one’s turn would take repeated practice for those with attention and/or hyperactivity difficulties. However, through the carer’s physical facilitation, children who were usually physically excluded could experience participation and have fun, while those with attention and behaviour difficulties got valuable practice at turn-taking, attentive looking and listening. The enjoyment of both children and carers was a factor supporting the sustainability of such games that support pre-verbal skills and inter-personal communications (Sameroff & Fiese 2000).
- Participatory group puzzle activities: These were implemented as another strategy to provide pre-verbal and early communication stimulation for attention, turn-taking and basic interaction for a whole, diverse group simultaneously. This took into account the scarcity of educational equipment such as pegboard puzzles and instead of wanting to give each child a puzzle to complete, each child would only get one piece to hold and await their turn as the carer moved the puzzle around and facilitated each child’s participation in the completion of the puzzle as a group effort – with much excited reinforcement of skills such as waiting for one’s turn and saying or indicating whose turn was next.

**Wrap-up with questions, self-evaluation and next steps:** At the end of each session, a brief time for wrap-up was introduced to meet the need for consolidation and to serve as a monitoring strategy. This was guided by two basic reflective questions, ‘*What did I learn today?’* and *‘What new game, activity or strategy will I try out in the coming week?’* These few minutes at the end of each session became an integral and valuable part of the on-site training and provided the speech therapist-trainer with insights into the strengths of the carers, training effectiveness and gaps that needed to be addressed as a priority in a subsequent session. It was informally observed that carers readily implemented these self-reflections and added to them during the week – as was evidenced by their responses in the opening challenge of the subsequent session.

## Challenges experienced

During the course of the training sessions in the sixteen special care centres, several challenges prompted adaptations in the training approach. The following occurred at least twice in separate situations:

- Trainer-initiated skills-sharing with carers resulted in carers becoming passive and/or withdrawing, and leaving the trainer to do the activity in question. Therefore, it was necessary to get buy-in from the carers in terms of what they felt were priority problems and what they had already tried.
- Individualised multi-step intervention programmes for individual children were difficult or impossible for the carers to implement in addition to their workload of basic ‘survival’ care. Therefore, activities that could benefit several children at once were more readily and sustainably applied.
- ‘Theoretical overload’: in several centres, there were written notes, posters and other forms of reminders from past on-site training interventions covering various topics on the walls. In most cases, these were not being implemented, and discreet enquiries elicited responses including, ‘There isn’t time to read it’ and ‘I don’t understand it’ and, in several observed situations, carers lacked the English language and/or literacy skill requirements of the written notes and posters. Therefore, written inputs were minimised.
- There had previously been piecemeal interventions by different therapists, with contradictory inputs. This was due to the precarious funding environment of NGOs and volunteer organisations working in the sector, and diverse organisations contributing short-term training inputs or one-off sessions. Unrelated or contradictory training inputs resulted in noticeably confused carers, and sometimes indifference or disinterest in training inputs. Therefore, the opening challenges and beginning with what the carers remembered from previous multidisciplinary training inputs helped.

## Discussion of results

Outcomes of the on-site communication training are best left in the words of the carers themselves. These were in response to the reflective question posed as a means of self-evaluation at the end of each session, ‘*What did I learn today?*’

A: ‘… I can talk to a child even if he cannot talk’
B: ‘… you must never underestimate them’
C: ‘… every child can be included in the games’
D: ‘… all of them enjoy ball games, even the buggy-ones’
E: ‘… every child has something to say’
F: ‘… even without speech, they can use their voice’
G: ‘… every child’s quality of life can grow’
H: ‘… if I enjoy myself, the children will have more fun.’

These responses helped to set the tone, level and pace of communication inputs during the training. From added observations and additional informal carer feedback, the following were identified as useful approaches that could be built on in future training:

- Building on the existing assets in each centre (for e.g., using existing song circles or naming rituals and adding value, as outlined above)
- Prioritising care for carers who were overstretched and under-resourced, and needed to feel understood and nurtured in order to care for the children with warmth and creativity
- A combination of dedicated sit-down information sharing and discussion, followed by hands-on approaches that took place in as natural a context as possible
- Opportunities for the carers to verbalise their needs as well as their problem-solving strategies and successes in terms of communication with the children in their care
- A brief, reflective self-review at the end of each session to consolidate and strengthen implementation of the learning experience. Language or terminology misunderstandings in the earlier tutorial section sometimes only surfaced during this time – but could at least then be resolved!

Some issues were identified and then addressed in response to needs or requests raised at specific special care centres with observable, continuing implementation during follow-up visits. These included:

- The replacement of obscure and non-transparent hand signs with appropriate, easily understood gestures, for example, a ‘salute’ greeting replaced the previously taught and non-transparent American Sign Language (ASL) ‘good + morning’ at one centre. This had immediate and lasting results. At least two children readily adopted the greeting and got immediate and friendly responses, even from uninformed members of the public. Six months later, the salute gesture was observed to be part of the spontaneous repertoire. This was used to effectively ‘connect’ with members of the community.
- Sportsmen’s wristbands, traditionally used to wipe perspiration off the forehead, were introduced as a means of drooling control for children who had a mild-moderate saliva control problem. With repeated reminders from carers, this form of control could replace less socially acceptable bibs in children who were physically able to wipe and cognitively able to grasp the instruction to do so – and in some cases increased inclusion in family and social events due to decreased ‘messiness associated with drooling’ were reported by carers who had implemented the ‘wristband training’ consistently with certain children. An increase in self-confidence (and upright posture!) was also reported in at least two cases.
- Inclusive games and song circles were seen to be sustainable as they helped the carers to provide enjoyable stimulation to a group and meet diverse goals with variably disabled children.

## Conclusions

On-site, hands-on training appears to be an effective strategy to support untrained and unskilled, but motivated carers in grassroots special care centres caring for groups of children with severe and profound disabilities, where poverty or other socio-environmental factors preclude parents from doing this in the home environment. While the ‘developed world’ model of low-ratio individual interventions by professional staff remains unrealistic, a long-term mentoring programme – ideally with a once monthly visit to each centre – would empower carers to implement valuable support for basic communication development (and enjoyment) for the children with severe and profound disabilities in their care.

### Implications and recommendations

The urgent and continued need for government level planning and service provision in terms of educational, training and therapy services for the much neglected population of children with severe or profound disabilities cannot be overemphasised. Simultaneously, however, a critical reflection of the activities carried out in this skills-sharing approach reinforces the community based rehabilitation (CBR) principle of building upon the assets or resources already in the community and indicates that a little goes a long way. The synergy of basic communication science, the will and motivation of the carers and the positive reinforcement of enhanced responsiveness and communication with children with severe and profound disabilities indicate some important possibilities.

Student training in the field of communication sciences and disorders (speech therapists) – as well as other rehabilitation disciplines – needs to address the preparation of students for the increasing needs related to severe and profound disabilities, especially in the field of intellectual disabilities. More alternatives to individual therapy models, including CBR strategies and training and, moreover, the enskilling of speech therapists to provide contextually appropriate carer training at grassroots levels need to be addressed both in undergraduate training and in continuing professional development inputs.

Furthermore, speech therapists’ collaboration with community-based rehabilitation workers in terms of carer training holds great potential for covering the extensive training needs newly opened up by the recent High Court ruling that children in special care centres will receive appropriate stimulation and/or educational inputs (Western Cape High Court 2011).

Formal research is needed to explore and evaluate aspects of such an on-site communication training approach. The need is crucial, to present an evidence base for more effective, efficient, relevant and sustainable strategies of empowering centre-based carers to provide optimal communication opportunities and support, for the children in their care to enjoy the basic human right of personal interaction and communicative participation, whatever the severity of their disability.

## Footnotes

1. The Care Dependency Grant is a government grant (administered by the South African Social Security Agency within the Ministry of Social Development) and is intended to support the primary carer of a severely disabled child under the age of 18. The reality is that the current amount of this grant (ZAR 1200 – approx. US$ 150) does not cover basic costs and many of these mothers still need to pursue employment to make ends meet.
